# Hospital and Institutional News

**Published:** 1919-03-22

**Authors:** 


### HOSPITAL AND INSTITUTIONAL NEWS.

### HEREDITY IN HOSPITAL LIFE.

FAMILY traditions are intertissued with the life of the voluntary hospitals, and they will continue to be so long as the voluntary system endures. Perhaps even afterwards they may do so, for is not the House of Commons rapidly becoming the hereditary House, as one by one the lords are shepherded into the city or crowded out by busy men who have made careers for themselves? Thinkers have argued before now that all professions will one day become hereditary; and from the time of the craft guilds and the workshops of the great painters to the modern solicitor and doctor whose sons adopt their fathers' professions, the tendency is a persistent and a natural one. But do we observe that, except in the case of the House of Commons, Government servants bring up their sons to the same calling? We should like statistics on the point, but we believe that the proportion would be found much smaller than in the case of the professions, the arts, and the trades. If this is so, then hospital family dynasties would not long survive perversion from the voluntary system to State or municipal control. We should then not be able to record as inspiring and far from strange a fact such as that the new President of the Liverpool Royal Infirmary, Mr. Arthur Crosthwaite, has now stepped into the post which was filled by his father in the year 1846. That is, we are proud to boast, seventy-three years ago. If a member of the same family becomes president in 1992, the voluntary system will have another century to its credit. The two traditions are interlocked.

### A DONOR WITH IDEAS.

THE gift of a piece of land by Mr. H. H. Wills to the Bristol Royal Hospital for Children and Women has been followed by a suggestion for its use, which deserves notice. Mrs. Wills, the wife of the donor, has asked her committee to consider whether the best use for the site would not be to build thereon accommodation for the surgical treatment of tuberculous joints. She declared that there was no provision for this class of case in Bristol, and that no modern appliances were available. On the other hand, she was informed that at least 100 children were now in need of such treatment, and in consequence of disease were excluded from school. The suggestion received no opposition, and was in fact endorsed by Dr. Cecil Williams and Mr. E. B. Wood. A donor with ideas is a donor worth having.

### SURPLUS EQUIPMENT FOR NEEDY HOSPITALS.

IN referring lately to the hospital sales which have been taking place on the closing of many auxiliary hospitals, we expressed the hope that the equipment thus disposed of would not find its way merely into the shops of local dealers. It is gratifying, therefore, to learn that a comprehensive scheme is now proposed. The proposal is to distribute at a moderate price the up-to-date surgical equipment to those hospitals which are most in need. How far inquiries, and how far the higgling of the market is to decide the destination of the goods is not stated. In connection with this is another report—namely, that temporary Red Cross hospital buildings, which can often be broken up into sections, should be rebuilt in districts where hospital accommodation is shortest. If this can be arranged many of those who are now proposing to build extensions or new cottage hospitals could with advantage use these structures until proper memorial hospitals can be built. Such a plan would give the districts actual institutions before public enthusiasm has had time to cool, and would thereby give great impetus to the appeals which are being issued. Both proposals, however, require prompt and skilful organisation.

### MORE WAR MEMORIALS HOSPITALS.

VERY naturally, the best war memorial that attracts those interested in hospitals is an improvement or extension of a hospital. And in many parts, those interested in this way are bringing forward schemes which are in harmony with the idea. At Gateshead, the committee of the Children's Hospital have approved a scheme to build an entirely new out-patient department and for the purpose appeal for £6,000. At Tunstall, a badly needed local Cottage Hospital is the war memorial proposed by an influential committee. It is sought to enlarge the Royal Hospital, Portsmouth, by the same method of raising money, while at Bradford a comprehensive scheme is presented which, if carried out, will alter the future of the Royal Infirmary, the Eye and Ear Hospital, the Children's Hospital, and the Bradford Hospital, and Convalescent Fund, as apparently an amalgamation, rather than a joint appeal, is in the minds of the promoters.

### THE DOMINIONS AND OUR HOSPITALS.

*St. Mary's Hospital Gazette* notes that a movement is on foot to obtain for men for the Dominions short terms of service as Residents in British hospitals before they sail for home. While regarding this effort with sympathy, the *Gazette* says, "The requirements and desires of such medical men should be held as strictly second to those of our own men. The desire to obtain clinical facilities while detained in England is perfectly natural, and, moreover, a compliment to British medicine. We think the fairer way of satisfying all parties is to reserve the first choice of vacant Resident posts for our own returned men, while at the same time increasing the opportunities for post-graduate work for our overseas visitors."

### LICE AND PUBLIC HEALTH.

RECENTLY published memoranda from the Local Government Board indicate that active steps will be taken against the great dangers possibly arising from the present-day combination of overseas disease, demobilisation, and louse-infested homes. The lessons of typhus and trench fever during the war have been only too obvious, and health departments must make the greatest endeavour to benefit from and appreciate them. A mixture—crude naphthalene 9 parts, and soft soap 1 part—is advised as the best repellant, while turpentine or paraffin oil is recommended for removal of nits from the hair. Clothing may be put into cold water, brought to the boil—and woollen articles should be ironed or placed, wrapped in brown paper, for half an hour in an oven of moderate heat. With regard to "itch" or scabies, a hot bath followed by the usual sulphur ointment is still considered as the most efficient method, but in all cases it must be remembered that mere notification is useless unless the means of prevention and cure are ready to hand and the situation, both at home and abroad, dealt with in a comprehensive manner. Public education is the keynote of success, and, judging by past experiences, the accepted ideas of personal cleanliness will, in many tenement homes, require considerable revision before any measure of improvement can be attained.

### HOSPITAL SAVED FROM DISASTER.

THE financial year just ended has been a trying one for many hospitals, among them the Royal Hamadryad Seamen's Hospital, Cardiff. The work done by the institution was greater than ever before, the total number of patients treated being 14,040, (as compared with 8,635 ten years ago), and the executive committee, in their report, candidly admit that it was the Jubilee Fund and a £750 grant from King George's Fund for Sailors which saved the hospital from disaster. The ordinary income would have been quite inadequate to meet the greatly increased cost of foodstuffs, drugs, and all requirements, and the hospital is to be congratulated on possessing a president in Lord Glanely who took the Jubilee Fund, which now amounts to £33,000 in hand, and made a success of it.

### NEWPORT'S FINANCIAL PROBLEM.

THE position of hospitals when the assistance from military or naval sources comes to an end was touched on in the report presented by the Directors of the Royal Gwent Hospital at Newport. At the conclusion of the last financial year the accumulated deficiency on the working account of the hospital had reached £3,442. Alderman Fred Phillips then offered to subscribe £442 if the Directors could raise the balance of £3,000 within three months. Mainly through the energy of Mr. W. H. Le Grand Chambers, Chairman of the Subscription Committee, the required amount was obtained, Mr. Phillips' cheque claimed, and the deficiency wiped off. Now, however, is the time for another campaign if the aim of the Directors to admit at once all cases requiring in-treatment is to be realised. The large sum received from the military authorities, it is pointed out, will not last much longer, and there is nothing to take its place. At Newport, as elsewhere, a permanent increase of the subscription income is the solution.

### A DOCTOR AND INFLUENZA.

ACCORDING to the *Manchester Guardian*, a certain doctor in Salford appears to have passed through a period of extraordinary activity and strain. A leaf from his day-book records that, in one day, private patients visiting or visited numbered 96, in addition to 100 on panels in Salford and Manchester. Patients first called at 7 A.M. From 8.45 A.M. to 1.15 P.M. he was out visiting at the rate of ten patients an hour. Resuming at 2 P.M., sixty to seventy consultations were given in his rooms, and the tour of visits then continued until 6 P.M. Half an hour later consultations restarted and went on until nearly 9 P.M., after which further calls had to be made on acute cases. As the practitioner is recorded to have stated, this kind of thing is not good for the brain, and if such experiences are falling to the lot of many, the recent Parliamentary announcement of increased medical demobilisation must be more than welcome.

### A BIRMINGHAM PAY HOSPITAL.

DR. ADDISON lately remarked in the House of Commons, in connection with the "Ministry of Health Bill," when it was reported, that so far as hospital accommodation goes "no one suffers more than the poorly paid middle classes." He added: "Voluntary hospitals cater for the poor, but the middle classes who cannot afford the fees of the expert, and do not want to be the recipients of charity, suffer through a lack of hospital facilities." Some good pay hospitals exist, but there are few of them, and some pretentious ones are very bad. In Birmingham there is a pay hospital called S. Chad's, which was built and equipped at an approximate cost of £25,000. There medical and surgical treatment, administered by consultants and under the observation of a qualified Resident Medical Officer, can be obtained "at a cost to the patient commensurate with his means." This phrase needs preciser definition. The founders include medical men drawn from the Consultant Staffs of the General and Special Hospitals in the city, and several men of business. We have received the following figures with reference to the patients, who are stated to be drawn from many spheres of life:—In-patients admitted: 1915, 488; 1916, 685; 1917, 840; 1918, 1,062. The hospital has been constructed and equipped for its work. It contains sixty private rooms, four small wards containing five beds each, two large open-air wards, two operating theatres, a pathological laboratory, an *x*-ray department, a dispensary, and a maternity department. The management of the hospital is conducted by a House Governor, Mr. D. J. Richards, a Matron, and a Resident Medical Staff, working under the direction of a Medical Committee and the Directors.

### AN UNUSUAL BATTLE CASUALTY.

AMERICA is famous for her records, and although she does not claim the following as one of them, yet we think it is an unusual instance of multiple wounds having a non-fatal termination. During the attack on the Hindenburg Line in 1918 Sergt.

Grimmer, A.E.F., formerly a New York chauffeur, was first partially gassed, and finally emerged from the fight with sixty-eight pieces of shell in his body, and three machine-gun bullet wounds in his right thigh. All the metal has now been removed. The figures are supplied by *The Medical Record*, New York. After receiving these wounds the man lay all day under shell fire, was carried four miles to a dressing-station, and then twenty miles by motor ambulance to a field hospital, a truly extraordinary record of endurance.

### THE GRAVE SCANDAL OF BETHNAL GREEN.

The Church of St. Martin's-in-the-Fields has for some time been doing well what many other places of worship have been doing badly. It has placed its pulpit, or its lectern, at the disposal of men of standing in the intellectual life of the day. In giving them half-an-hour, usually during London's luncheon hour, it has done good service to busy but awakened people, and incidentally filled its church. The speakers are often laymen. The philosopher, the headmaster, the man with a subject worthy of his opportunity at this famous church, has had his chance. Religion has not been cheapened: the cackle which often passes for controversy has not been heard. The work has been little advertised beyond what posters on the church walls could do to inform the passer-by of coming addresses. For this reason perhaps it has had the reward which is *not* a *réclame*. But good work cannot continue indefinitely in the quietness proper to its beginnings as the following facts show. On February 22 last Lieut.-Colonel W. J. Lewis, who is the Mayor of Bethnal Green, was the speaker. He was original enough to find his subject in the present condition of his borough, and Mr. Councillor Edmonds with the Bishop of Stepney followed him. The story told that day attracted less attention in the ruck of newspapers than their beauty competitions or their football news. We therefore ventured to reproduce the substance of Councillor Edmonds' indignant exposure, beside the facts disclosed by Colonel Lewis, and called it, with due restraint, "The Grave Scandal of Bethnal Green" (The Hospital, March 1, page 479). Exactly thirteen days later Colonel Lewis was summoned by Her Majesty to Buckingham Palace, because, as Printing House Square put it, "the attention of the Queen was attracted by an account of his address," and Her Majesty expressed a wish to see its author. After a discussion and an examination of plans had taken place the Queen is declared to have said: "It seems pretty clear to me that when I have visited the poorer districts I have been taken mainly to the highways and not the byways." But if the entire Press had served the Throne as loyally as the Throne daily serves the people, the Press would not have "the responsibility of silence" on its conscience.

### THE SALARIES OF TOWN CLERKS.

Every little while, even such times of retrenchment as these should be, one notices paragraphs in provincial newspapers announcing that the clerk of such and such a town or city has had his salary raised from £900 to £1,000 per annum, or from £1,000 to £1,250. There is usually some little opposition, but the matter seems to have been well set in train beforehand, for it almost invariably goes through. It is hard to see its justice, however. A town clerk is almost always a barrister too, and if he thinks his remuneration insufficient it is perfectly open to him to try his powers in competition with others at the bar. Moreover, every one knows that a clerk of a prosperous town is exceedingly well placed for having a share in various profitable local undertakings. On the other hand, there are dozens of medical men, whose education has cost them more than a barrister's, who are surely at least equally beneficial to the community, whose work cannot be delegated to subordinates, and is sometimes night and day together, who have expenses like motoring and drug bills to meet, and yet whose gross takings are much less. If ever it come to pass that medical members of Parliament increase at the expense of legal ones this is a matter for them to look to. At the next election it might well form a subject of preliminary interrogation for medical candidates.

### THE FUNCTIONS OF A FOOD SUPERVISOR.

As will be seen from our advertisement columns, the General Hospital, Birmingham, is seeking candidates for the post of food supervisor at a salary of £130 a year, with board, residence, uniform, and laundry. The officer will be required to take entire charge of, and be responsible for, the purchase, preparation, and distribution of food. As one of the advertised emoluments takes the form of uniform it is indicated that the post is one to be held by a woman. The functions of a food supervisor are presumably similar to those usually carried out by the housekeeper in a large hospital except that supervision of domestic staff other than that employed in the preparation and distribution of food is allocated to another officer. St. Bartholomew's Hospital led the way by the appointment of a food controller over a year ago. It would be interesting, in view of the vital importance of this branch of hospital work, to learn if similar appointments have been made elsewhere.

### A NORFOLK HOSPITAL SCHEME.

The Board of Management of the Norfolk and Norwich Hospital are issuing an appeal for £30,000 in order to carry out a scheme for converting an old part of the hospital into a new and well-equipped orthopædic department. The project is not to be launched as a form of war memorial; it is expressly stated that it is not intended to challenge comparison with other enterprises. Before the war about 150 patients weekly were under treatment in the electro-therapeutic department; now the number is nearly 500, of whom 200 are discharged soldiers. The existing accommodation is hopelessly inadequate. The scheme aims at the complete reorganisation of the old wing of the hospital, which was built 150 years ago and has never been adapted to modern needs.

### £40,000 FOR WOMEN'S HOSPITAL.

In little more than twelve months the friends and supporters of the Elizabeth Garrett Anderson Hospital, Euston Road (formerly the New Hospital for Women), have raised nearly £40,000 of the £50,000 required for the endowment fund in memory of the late Mrs. Garrett Anderson. This success is the more noteworthy from the fact that most of the money has been obtained from women's colleges, girls' schools, and different groups of women representing all classes and many professions. Two beds have been endowed with money collected among former patients, and this affords striking testimony to the popularity of the institution.

### HOSPITAL SECRETARY AS AUDITOR.

Mr. Gilbert D. Shepherd, F.C.A., senior partner in the firm of Shepherd, Howell & Co., chartered accountants, Cardiff, is gaining a varied experience in hospital accountancy. For eight years Mr. Shepherd has been auditor to King Edward VII's Hospital, Cardiff, for five years auditor to the Cardiff and District Hospital Society, and for two years secretary of the Prince of Wales' Hospital for Limbless Sailors and Soldiers, Cardiff. Mr. Shepherd has now been appointed auditor to the Cardiff City Mental Hospital, Whitchurch, Glam., where there are over 1,000 beds.

### BARRASFORD SANATORIUM'S FINANCES.

Confronted with a deficit of £567 on the year's work, and desirous to reduce the maintenance charges if possible to two guineas a week, the Committee of Barrasford Sanatorium, Newcastle, decided to begin the new year by doubling its accommodation. To be precise, we should say that the Committee determined to raise the number of beds to eighty-five, which, it was thought, could be done by reorganising the institution, and consequently for a comparatively small capital sum. The plan rests upon the estimate that with eighty-five beds continuously occupied, the fees can be reduced to two guineas. On New Year's Day, 1918, there were thirty-eight patients residing at Barrasford. On December 31 last there were forty-nine. It may be inferred from this that eligible patients can rarely, if ever, afford more than two guineas a week, and consequently that when fees are higher the beds are not fully occupied. By increasing the beds it is hoped to reduce the fees. What a perfect instance of the hollowness of the old argument that a hospital in difficulties can save money by closing its beds! We wish well to the suggestion, and to the imaginative and public-spiritedness which prompted it. How far sanatorium superintendents interest themselves in finance is perhaps uncertain, but it would be interesting to know Dr. Goodwin's opinion on the likelihood of success for the new policy.

### ONE THOUSAND POUNDS FOR A SHILLING.

A great movement is progressing in Sydney to celebrate the fiftieth anniversary of the Royal Prince Alfred Hospital, and a jubilee fund is the subject of an appeal, the total aimed at being £40,000. The object of the fund is to provide in the hospital improved apparatus and conditions for the treatment and comfort of returned soldiers, and to increase accommodation and equipment generally. It is proposed to devote part of the fund to extinguish a debt of £20,000 that has accrued since the war began. One of the means of raising money is a huge lottery or art union in which the first prize is a £1,000 War Bond, and objects of art which many of the purchasers of the shilling tickets will hope to acquire. There are many other prizes, the total value being stated at £1,805, including household furniture, a typewriter, a sewing-machine, and a five-stone diamond ring. Those who "turned down" the Red Cross pearl necklace lottery idea will no doubt receive the intelligence with uneasiness, but, human nature being what it is, we expect that the great hospital of Sydney will reap a rich harvest by means of this mild gamble.

### THE LAST OF THE LINEN CASE.

Mr. E. Pitts Fenton, clerk to the Bermondsey Board of Guardians, has resigned after 41 years connection with the Poor Law. For the past 27 years he has been clerk to the Bermondsey Board of Guardians. Latterly he has been the central figure in a prolonged inquiry held by Mr. J. S. Oxley, an inspector of the Local Government Board, into extensive purchases of drapery, without, it was maintained, either the knowledge or consent of the guardians. After lengthy proceedings, the Local Government Board issued a report severely criticising the clerk and the guardians. The guardians are now selling a large portion of this drapery, after reserving for themselves sufficient stock to last them well into next year, and it is anticipated that there will be a fair profit over the transaction. The departure of Mr. Fenton may remove the friction that has existed in Bermondsey over the administration of the Poor Law in the borough.

### THIS WEEK'S DRUG MARKET.

The steady downward movement in prices continues, and so far as a fairly long list of drugs is concerned it is safe to assume that this movement has by no means reached an end. Of course, as time goes on the downward tendency may be slower, as the margin between present values and the possible lowest level becomes narrower and narrower. Bromides generally speaking are in much the same position as last week, although lower figures are quoted for the ammonium salt. Aspirin continues to tend downwards. Benzoic acid is again cheaper. Formaldehyde is in better supply and offers at lower rates. Menthol is quieter, and the upward tendency in price seems to have been checked for the time being. Among other articles quoted at a lower rate may be mentioned creosote carbonate, guaiacol carbonate, methyl, salicylate, citronella oil, resorcin, and theobromide. Potassium permanganate is offered more freely at lower prices. Sugar of milk is getting more plentiful and the price is tending downwards. Salicylate of soda is again lower in price. Cloves are cheaper and clove oil may again follow suit.

